# Aberrant Expression of COT Is Related to Recurrence of Papillary Thyroid Cancer

**DOI:** 10.1097/MD.0000000000000548

**Published:** 2015-02-13

**Authors:** Jandee Lee, Seonhyang Jeong, Jae Hyun Park, Cho Rok Lee, Cheol Ryong Ku, Sang-Wook Kang, Jong Ju Jeong, Kee-Hyun Nam, Dong Yeob Shin, Eun Jig Lee, Woong Youn Chung, Young Suk Jo

**Affiliations:** From the Department of Surgery (JL, CRL, SWK, JJJ, KHN, WYC); Department of Internal Medicine, Severance Hospital, Yonsei Cancer Center, Yonsei University College of Medicine, Seoul (SJ, CRK, DYS, EJL, YSJ); and Department of Surgery, Yonsei University Wonju College of Medicine, Kangwon (JHP), Korea.

## Abstract

Aberrant expression of Cancer Osaka Thyroid Oncogene mitogen-activated protein kinase kinase kinase 8 (COT) (MAP3K8) is a driver of resistance to B-RAF inhibition. However, the de novo expression and clinical implications of COT in papillary thyroid cancer (PTC) have not been investigated.

The aim of this study is to investigate the expression of A-, B-, C-RAF, and COT in PTC (n = 167) and analyze the clinical implications of aberrant expression of these genes.

Quantitative polymerase chain reaction (qPCR) and immunohistochemical staining (IHC) were performed on primary thyroid cancers. Expression of COT was compared with clinicopathological characteristics including recurrence-free survival. Datasets from public repository (NCBI) were subjected to Gene Set Enrichment Analysis (GSEA).

qPCR data showed that the relative mRNA expression of *A-*, *B-*, *C-RAF* and *COT* of PTC were higher than normal tissues (all *P* < 0.01). In addition, the expression of COT mRNA in PTC showed positive correlation with A- (*r* = 0.4083, *P* < 0.001), B- (*r* = 0.2773, *P* = 0.0003), and C-RAF (*r* = 0.5954, *P* < 0.001). The mRNA expressions of A-, B,- and C-RAF were also correlated with each other (all *P* < 0.001). In IHC, the staining intensities of B-RAF and COT were higher in PTC than in normal tissue (*P* < 0.001). Interestingly, moderate-to-strong staining intensities of B-RAF and COT were more frequent in B-RAF^V600E^-positive PTC (*P* < 0.001, *P* = 0.013, respectively). In addition, aberrant expression of COT was related to old age at initial diagnosis (*P* = 0.045) and higher recurrence rate (*P* = 0.025). In multivariate analysis, tumor recurrence was persistently associated with moderate-to-strong staining of COT after adjusting for age, sex, extrathyroidal extension, multifocality, T-stage, N-stage, TNM stage, and B-RAF^V600E^ mutation (odds ratio, 4.662; 95% confidence interval 1.066 − 21.609; *P* = 0.045). Moreover, moderate-to-strong COT expression in PTC was associated with shorter recurrence-free survival (mean follow-up duration, 14.2 ± 4.1 years; *P* = 0.0403). GSEA indicated that gene sets related to B-RAF-RAS (*P* < 0.0001, false discovery rate [FDR] *q*-value = 0.000) and thyroid differentiation (*P* = 0.048, FDR *q*-value = 0.05) scores were enriched in lower COT expression group and gene sets such as T-cell receptor signaling pathway and Toll-like receptor signaling pathway are coordinately upregulated in higher COT expression group (both, *P* < 0.0001, FDR *q*-value = 0.000).

Aberrant expression of A-, B-, and C-RAF, and COT is frequent in PTC; increased expression of COT is correlated with recurrence of PTC.

## Abstract 2

Supplemental Digital Content is available in the text

## INTRODUCTION

Patients with papillary thyroid cancer (PTC) generally have a relatively indolent clinical course and favorable therapeutic outcomes,^1^ although persistent or recurrent PTC occurs in 10% to 15% of cases.^2^ Management of persistent or recurrent PTC consists of surgical resection followed by radioactive-iodine (RAI) therapy.^3,4^ However, a significant proportion of persistent or recurrent PTC is not amenable to surgical resection and shows RAI refractoriness.^4^

The RAS-RAF-mitogen-activated protein kinase (MAPK) pathway is one of the best characterized signal pathways to transduce a signal from a receptor on the cell surface to the nucleus of the cell.^5^ In this conserved signaling pathway, RAF proteins such as A-, B-, and C-RAF are activated by RAS and then lead to activation of the dual-specific protein kinases MEK1/2 (mitogen-activated protein kinase kinase, MAPK kinase) and subsequently ERK1/2 (extracellular signal-regulated kinase, ERK, MAPK).^5^ Although all 3 RAF proteins regulate ERK signal pathway, the individual RAF isoforms can be differentially regulated in cell type-specific or context-dependent manner.^6–8^ In addition, the RAF is forms have strikingly different phosphorylation sites.^5^ Recently, A-RAF has been reported to act as a scaffold to stabilize B-RAF:C-RAF heterodimers, whereas A-RAF dimerization also promotes MAPK activation.^9,10^ Besides 3 RAF is forms, Cancer Osaka Thyroid Oncogene mitogen-activated protein kinase kinase kinase 8 (COT) (MAP3K8), a serine/threonine kinase, was shown to play a role in the MAPK activation.^11^ To explain the regulatory mechanism of MAPK activation by COT, it has been suggested that COT is able to phosphorylate MEK-1.^12^

In fact, PTC is the result of the abnormal activation of RAS-RAF-MAPK signal pathway induced by RET/PTC rearrangement, Ras mutations, or B-RAF^V600E^ mutation.^4^ Following the discovery that the B-RAF^V600E^ mutation is present in a high proportion of many human cancers,^13^ several novel targeted agents were developed for B-RAF^V600E^-positive cancers.^14^ Because the incidence of the B-RAF^V600E^ mutation in PTC is high (40%–80%), these new agents were considered promising therapeutic modalities.^15,16^ Preclinical studies indicated the dependency of B-RAF^V600E^ tumors on MAPK signaling cascade, whereas the efficacy of both RAF and MEK inhibitors has been demonstrated in several clinical trials.^17–19^ However, de novo and acquired resistance to these agents has since emerged as a new therapeutic obstacle.^20,21^

Mechanisms of resistance to RAF inhibitors can be divided into two categories according to the dependency on RAF dimerization.^22,23^ In the first category, mutations in NRAS such as NRAS Q61, the p61BRAF^V600E^ splice variant, and C-RAF overexpression are involved in a mechanism that is dependent on RAF dimerization. The p61B-RAF^V600E^ splice variants lacking the RAS-binding domain can dimerize in a RAS-independent manner and generate MEK-ERK signal propagation. NRAS mutation and increased expression of C-RAF can also increase RAF dimerization which is insensitive to RAF inhibitor.^24–26^ In the second category, aberrant expression of COT or MEK mutation functions independently of RAF dimerization.^11^ Based on a functional genomic approach, COT can generate resistance to RAF inhibitors by MEK dependent mechanisms.^11,23^ While the newer targeted agents are often administered to patients with persistent or recurrent PTC,^27^ the incidence of *de novo* or acquired drug resistance in these patients has not been determined with certainty. Nonetheless, the outcomes in patients with PTC following administration of RAF (Sorafenib) or MEK inhibitor (Selumetinib, AZD6244, ARRY-142886) are generally poor compared with other cancers such as melanoma.^28–30^

In this study, we investigated the expression status of A-, B-, C-RAF, and COT mRNA in PTC with respect to that in matched normal thyroid tissues and analyzed the relationship between COT expression and that of RAF paralogues to investigate the presence of de novo drug resistance mechanisms and understand the clinical implications of aberrant expression of these genes.

## METHODS

### Subjects and Clinical Data

This study enrolled 167 patients (34 male and 133 female) undergoing total thyroidectomy with or without neck node dissection followed by radioactive iodine ablation for management of classical PTC from January 1987 to December 2002 at Severance Hospital, Seoul, South Korea. The study subjects showed no visible remnant in the first Diagnostic ^131^I whole body scan (WBS) with following thyroid hormone withdrawal (THW) performed 6 to 12 months after remnant ablation. The sample size was calculated by Web-based Sample Size/Power Calculations (http://www.stat.ubc.ca). Patient information and clinicopathological parameters were analyzed retrospectively; the overall median follow-up time was 14.2 ± 4.1 years. During this time, recurrence was diagnosed by: histopathologic diagnosis of clinically suspicious lymph node identified by neck ultrasound or physical examination (n = 23, 82.1%); newly detected lesion in ^131^I diagnostic WBS, 18-Fluoro-deoxyglucose positron emission tomography (FDG PET/CT) or chest computed tomography (CT) (n = 5, 17.9%) performed due to patient's serum thyroglobulin ≥2 μg/L with gradual increase following THW. Tissue samples were taken from the central area of the tumor and from contralateral histologically normal tissue. On histological examination, cellularity was >90% in all primary PTCs. All protocols were approved by the institutional review board of Severance Hospital.

### RNA Isolation and Real-rime PCR

Total RNA was extracted using Trizol reagent (Invitrogen, Carlsbad, CA, USA), and complementary DNA (cDNA) was prepared from total RNA using M-MLV reverse transcriptase (Invitrogen) and oligo-dT primers (Promega, Madison, WI, USA). Quantative RT-PCR (qRT-PCR) was performed on cDNA using the QuantiTect SYBR Green RT-PCR Kit (Qiagen, Valencia, CA, USA) with the following primers: A-RAF, 5′-CCT GGC GTT CTG TGA CTT CTG-3′ and 5′-CGG TTG GTA CTC ATG TCA ACA C-3′; B-RAF, 5′-GTG GAT GGC ACC AGA AGT CA-3′ and 5′-AGG TAT CCT CGT CCC ACC AT-3′; C-RAF, 5′-GGG AGC TTG GAA GAC GAT CAG-3′ and 5′-ACA CGG ATA GTG TTG CTT GTC-3′; COT, 5′-ATG GAG TAC ATG AGC ACT GGA-3′ and 5′-GCT GGC TCT TCA CTT GCA TAA AG-3′; interferon, gamma (IFNG), 5′-TCG GTA ACT GAC TTG AAT GTC CA-3′ and 5′-TCG CTT CCC TGT TTT AGC TGC-3′; lymphocyte-specific protein tyrosine kinase (LCK), 5′-TCT GCA CAG CTA TGA GCC CT-3′ and 5′-GAA GGA GCC GTG AGT GTT CC-3′; CD247, 5′-GGC ACA GTT GCC GAT TAC AGA-3′ and 5′-CTG CTG AAC TTC ACT CTC AGG-3′; chemokine (C-X-C motif) ligand 10 (CXCL10), 5′-GTG GCA TTC AAG GAG TAC CTC-3′ and 5′-TGA TGG CCT TCG ATT CTG GAT T-3′; chemokine (C-X-C motif) ligand 11 (CXCL11), 5′-GAC GCT GTC TTT GCA TAG GC-3′ and 5′-GGA TTT AGG CAT CGT TGT CCT TT-3′; toll-like receptor 7 (TLR7), 5′-CAC ATA CCA GAC ATC TCC CCA-3′ and 5′-CCC AGT GGA ATA GGT ACA CAG TT-3′; toll-like receptor 8 (TLR8), 5′-GAC TAC AGG AAG TTC CCC AAA C-3′ and 5′-ATA CCG GGA TTT CCG TTC TGG-3′; glyceraldehyde-3-phosphate dehydrogenase (GAPDH), 5′-GGA GCG AGA TCC CTC CAA AAT-3′ and 5′-GGC TGT TGT CAT ACT TCT CAT GG-3′. qRT-PCR experiments were repeated 3 times, and each experiment was performed in triplicate.

### DNA Isolation and Dideoxysequencing

Genomic DNA from formalin-fixed, paraffin-embedded tissue specimens was prepared from five 10-μm sections after microdissection. In the case of cancers, paraffin-embedded thyroid tissue specimens had >90% tumor cells. Genomic DNA was isolated using the EZ1 DNA Tissue Kit (Qiagen, Chatsworth, CA, USA). Exon 15 of the *BRAF* gene was amplified by PCR using standard conditions (95°C × 5 min; 94°C × 30 s, 58°C × 30 s,72°C × 30 s, for 32 cycles; 70°C × 10 min) and the following primers: forward 5′-ATG CTT GCT CTG ATA GGA AA-3′ and reverse 5′-ATT TTT GTG AAT ACT GGG GAA-3′. The amplified products were purified with the MinElute PCR Purification Kit (Qiagen) and were then sequenced on an ABI PRISM 3730XL automated capillary DNA Sequencer using the BigDye Terminator Cycle Sequencing Ready Reaction Kit (Applied Biosystems, Foster City, CA, USA).

### Western Blot Analysis and Immunohistochemical Staining

Western blot analysis was performed according to standard methods with commercially available antibodies: A-RAF rabbit polyclonal antibody (#4432, Cell Signaling, Danvers, MA, USA), B-RAF rabbit polyclonal antibody (sc-9002, Santa Cruz Biotechnology, Inc., Dallas, Texas, USA), C-RAF rabbit polyclonal antibody (#9422, Cell Signaling, USA) and COT rabbit polyclonal antibody (sc-720, Santa Cruz Biotechnology) and anti-β-Actin Antibody (#4967, Cell Signaling). Immunohistochemical staining (IHC) for B-RAF and COT was performed in 167 cases of PTC and matched normal tissues. Briefly, 4-μm tissue sections were heated at 60°C, deparaffinized in xylene, and hydrated in a graded series of alcohol. Antigen retrieval was performed by microwaving in citrate buffer for 10 min. Endogenous peroxidase activity was inactivated by incubation in 3% hydrogen peroxide for 10 min. Nonspecific binding sites were blocked by incubating in 10% normal goat serum diluted with phosphate-buffered saline. Tissue sections were then incubated with primary antibodies: B-RAF (sc-9002) or COT rabbit polyclonal antibody (sc-720) for 60 min at room temperature. All sections were sequentially treated with biotinylated anti-rabbit immunoglobulin for 30 min, peroxidase-labeled streptavidin for 30 min, and diaminobenzidine in the presence of hydrogen peroxide. Controls were incubated with PBS in place of primary antibody, and no positive staining was observed in any case. In addition to negative controls, sections of human fallopian tube tissues were used as a positive control for B-RAF and human small intestine tissues as a positive control for COT. Staining was scored as follows: 1, no staining; 2, weak or focal staining; 3, moderate staining in most cells; and 4, strong staining in most cells. To support our IHC-P (Immunohistochemistry-Paraffin Embedded Tissues) data, we reviewed the representative images of IHC-P for A-, B-, C-RAF and COT from Human Protein Atlas program (http://www.proteinatlas.org/).

### Gene Set Enrichment Analysis of COT-Correlated Genes

Microarray data from the Gene Expression Omnibus (GEO) of NCBI (Data Set Record GSE33630) were subjected to gene set enrichment analysis (GSEA).^31^ Genes strongly correlated to *COT* were verified by qRT-PCR using cDNA from our subjects.

### Statistical Analysis

Statistical analysis was carried out using either SPSS version 18.0 for Windows (IBM Corporation, Armonk, NY, USA) or GraphPad Prism (GraphPad Software, Inc, San Diego, CA, USA). Data are presented as the mean ± standard deviation. All *P* values are 2-sided.

## RESULTS

### Increased Expression of A-, B-, C-RAF and COT mRNA in PTC

**FIGURE 1:**
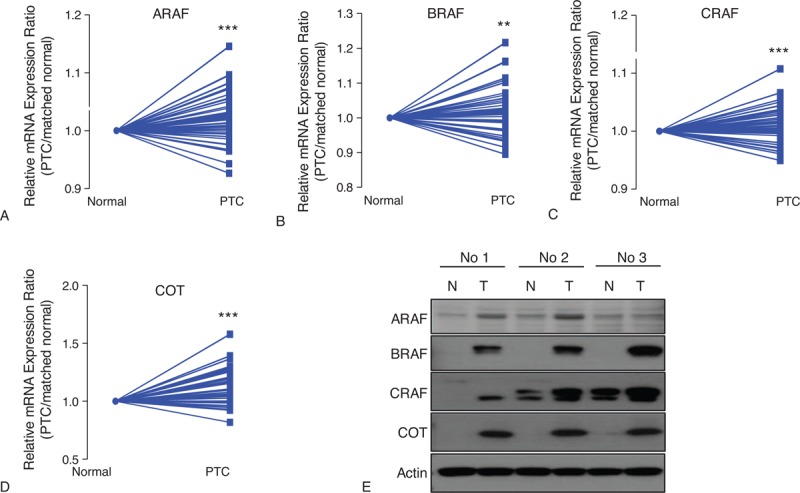
Comparison of relative mRNA expression ratio (PTC/matched normal thyroid tissue) and protein expression for A-RAF (A), B-RAF (B), C-RAF (C), and COT (D). Relative expression was calculated using the StepOne™ Real-time PCR System (Applied Biosystems, Foster City, CA, USA). 2^ΔCtn^ = 2^CtGAPDH-Cttarget^ calculation was used for relative mRNA expression value. Relative mRNA expression ratio was calculated by relative mRNA expression value of PTC/that of matched normal thyroid tissue (n = 135). Average ratios were compared with the paired *t* test. Data are average mean values. ^∗∗^*P* < 0.01, ^∗∗∗^*P* < 0.001. All *P* values are 2-sided. (E) Representative western blot analysis to compare protein expression between PTC and matched normal thyroid tissues (n = 3). All experiments were repeated 3 times, and each experiment was performed in triplicate. COT = Cancer Osaka Thyroid Oncogene mitogen-activated protein kinase kinase kinase 8, PTC = papillary thyroid cancer.

**FIGURE 2:**
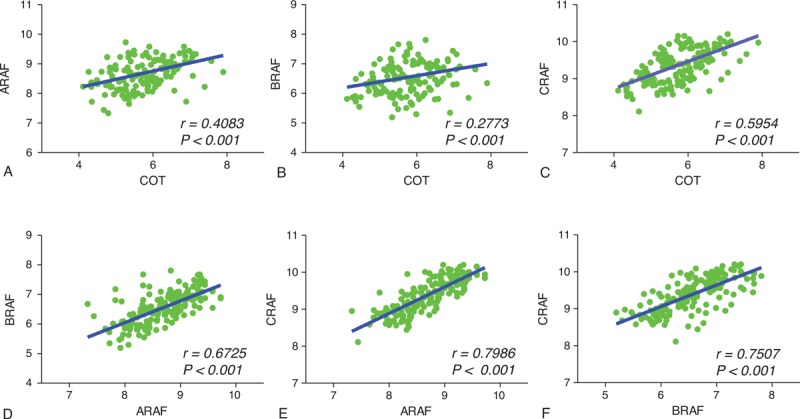
Correlation analysis of COT with A-, B-, and C-RAF in PTC. The relationship of relative mRNA expression values of COT (MAP3K8) with that of A-, B-, and C-RAF (n = 135). The relationship between 2 groups was analyzed by Pearson correlation analysis. *r* = Pearson correlation coefficient. COT = Cancer Osaka Thyroid Oncogene mitogen-activated protein kinase kinase kinase 8, PTC = papillary thyroid cancer.

To investigate the expression of A-, B-, C-RAF, and COT in PTC, we first performed qPCR using mRNA derived from primary PTC and contralateral matched normal thyroid tissues. Excluding 32 cases presenting poor isolated mRNA quality, we conducted mean comparisons using a paired *t*-test (n = 135). As shown in Figure 1 A–D, the relative mRNA expression of A-, B-, C-RAF and COT were higher than normal tissues. Supporting our qPCR data, western blot analysis showed that A-, B-, C-RAF and COT expressions were increased in PTC compared with matched normal tissues (Figure 1E). Furthermore, expression of COT mRNA significantly correlated with that of *A-* (*r* = 0.4083, *P* < 0.001), *B-* (*r* = 0.2773, *P* = 0.0003), and *C-RAF* (*r* = 0.5954, *P* < 0.001) (Figure 2A–C. The mRNA expression of A-, B-, and C-RAF correlated with each other (Figure 2D–F). Interestingly, the mRNA expressions of A-, B-, C-RAF and COT were higher in BRAF^V600E^-positive PTC compared with BRAF^V600E^-negative PTC (Supplementary Fig. 1, http://links.lww.com/MD/A207).

### Expression of BRAF and COT Proteins in PTC

**FIGURE 3:**
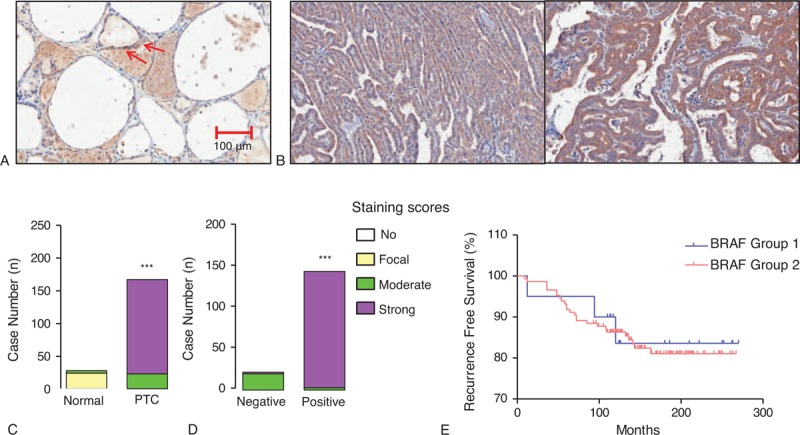
Relation of B-RAF expression with B-RAF^V600E^ mutation and recurrence-free survival. (A) Representative image of IHC-P using anti-B-RAF antibody in normal thyroid tissue (original magnification ×200). Arrows indicated focal staining intensity of B-RAF. (B) Representative images of B-RAF IHC-P in PTC (original magnification ×200). (C) Comparison of B-RAF expression between normal thyroid tissues and PTC. (D) B-RAF expression status according to the absence or presence of B-RAF^V600E^ mutation. IHC staining was scored as described in the Methods (n = 167). Group comparisons were performed by linear-by-linear association. (E) Kaplan–Meier estimates of recurrence-free survival according to BRAF expression. Group 1 indicates patients with PTC showing moderate staining of BRAF; Group 2, indicates strong staining intensity. IHC = immunohistochemical staining, PTC = papillary thyroid cancer.

**FIGURE 4:**
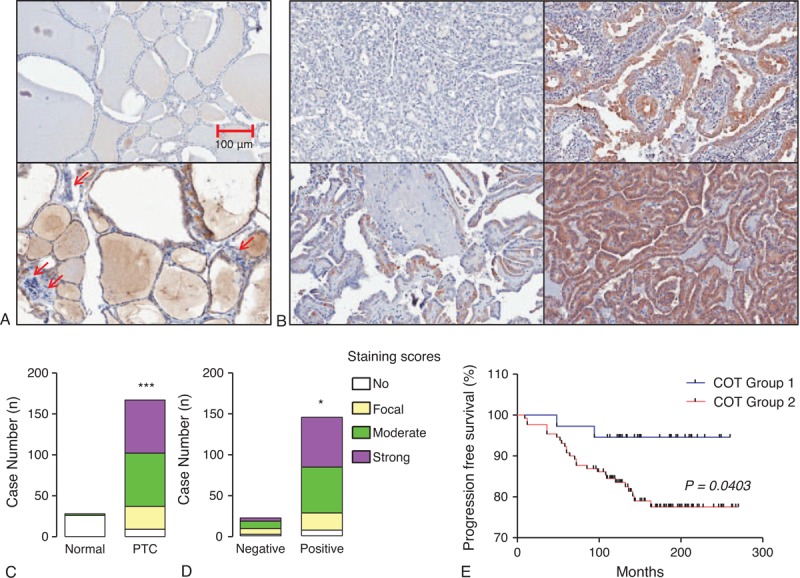
Relation of COT expression with BRAF^V600E^ mutation and recurrence-free survival. (A) Representative images of IHC-P using anti-COT antibody in normal thyroid tissue (original magnification ×200). Arrows indicated lymphocytic infiltration around thyroid follicles. (B) Representative IHC in PTC ranging from no staining to strong staining of COT (original magnification ×200). (C) Comparison of COT expression between normal thyroid tissues and PTC. (D) COT expression status according to the absence or presence of B-RAF^V600E^ mutation. IHC staining was scored as described in the Methods (n = 167). Group comparisons were performed by linear-by-linear association. (E) Kaplan–Meier estimates of recurrence-free survival according to COT expression. Group 1 indicates patients with PTC showing no staining or focal staining of COT; Group 2, indicates moderate to strong staining intensity. COT = Cancer Osaka Thyroid Oncogene mitogen-activated protein kinase kinase kinase 8, IHC = immunohistochemical staining, PTC = papillary thyroid cancer.

To validate our qPCR data and western blot analysis, IHC was performed using formalin-fixed, paraffin-embedded thyroid tissue blocks (number of tumor samples = 167). In fact, we tried to perform IHC-P for A-, B-, C-RAF and COT. However, because we could observe no staining intensity of A-RAF and focal or weak nuclear staining intensities of C-RAF (as presented in Human Protein Atlas, Supplementary Fig. 2, http://links.lww.com/MD/A207), we analyzed the results of IHC-P data for B-RAF and COT. Twenty-four cases out of 28 normal thyroid tissues showed only focal staining for B-RAF and remaining 4 cases showed moderate staining, although PTC showed moderate-to-strong staining intensities (Figure 3A and B). PTC showed significantly higher staining of B-RAF compared with normal thyroid tissues (*P* < 0.001, Figure 3C). Interestingly, expression of B-RAF was more frequently detected in B-RAF^V600E^-positive PTC (*P* < 0.001, Figure 3D). However, statistical analysis to investigate the correlation of B-RAF expression status with clinicopathological parameters did not show any significant implication of B-RAF expression on clinical parameters such as Kaplan–Meier analysis of recurrence-free survival (Figure 3E). In the case of COT, 26 cases out of 28 normal thyroid tissues did not show any staining intensity (Figure 4A, upper panel) whereas remaining 2 cases presented moderate staining intensity (Figure 4A, lower panel). Interestingly, these 2 cases of normal thyroid tissues with moderate COT staining showed lymphocytic infiltration around thyroid follicles (arrows). Although PTC showed various staining intensities of COT that ranged from no staining to strong staining (Figure 4A and B), our analysis of group comparison indicated that PTC showed significantly higher staining of COT compared with normal thyroid tissues (*P* < 0.001, Figure 4C). Interestingly, aberrant expression of COT was more frequently detected in B-RAF^V600E^-positive PTC (*P* = 0.013, Figure 4D).

### Clinical Implications of COT Expression in PTC

Based on IHC data that indicated higher expression of COT in PTC, statistical analyses were performed to investigate possible correlations with clinicopathological parameters. Interestingly, aberrant expression of COT (moderate-to-strong staining) was related to old age at initial diagnosis (*P* = 0.045, Table 1) and higher prevalence of B-RAF^V600E^ mutation (*P* = 0.023). Moreover, the recurrence rate of PTC was significantly higher in PTC showing moderate-to-strong staining (*P* = 0.025). In multivariate analysis, tumor recurrence was associated with moderate-to-strong staining of COT after adjusting for age, sex, extrathyroidal extension, multifocality, T-stage, N-stage, TNM, and B-RAF^V600E^ mutation (odds ratio [OR] 4.662; 95% confidence interval [CI] 1.066–21.609; *P* = 0.045, Table 2). Furthermore, Kaplan–Meier analysis revealed that moderate-to-strong COT expression in PTC was associated with shorter recurrence-free survival (mean follow-up duration; 14.2 ± 4.1 years, *P* = 0.0403, Figure 4E), strongly suggesting that aberrant expression of COT is associated with recurrence of PTC.

**TABLE 1:**
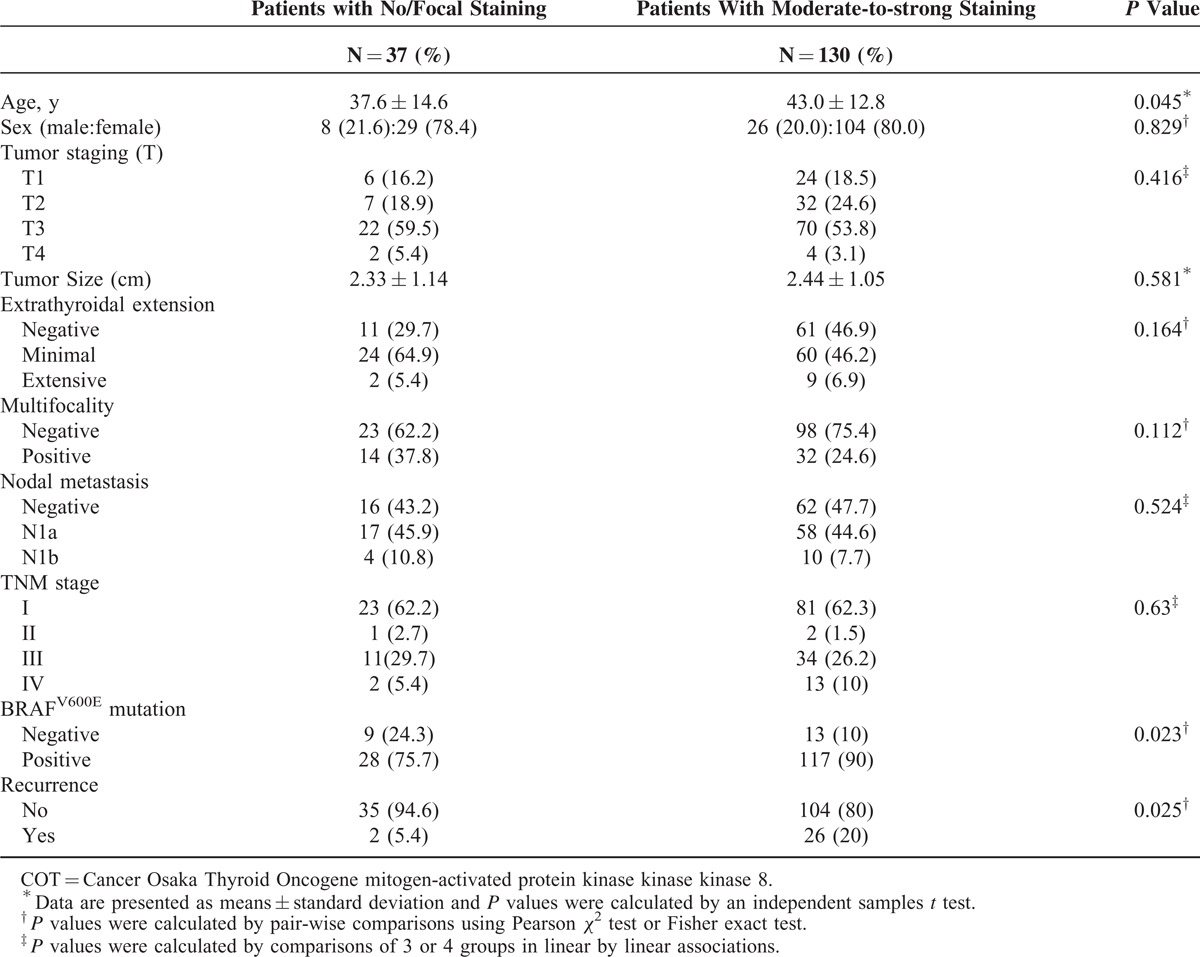
Clinicopathological Characteristics According to COT Expression Status

**TABLE 2:**
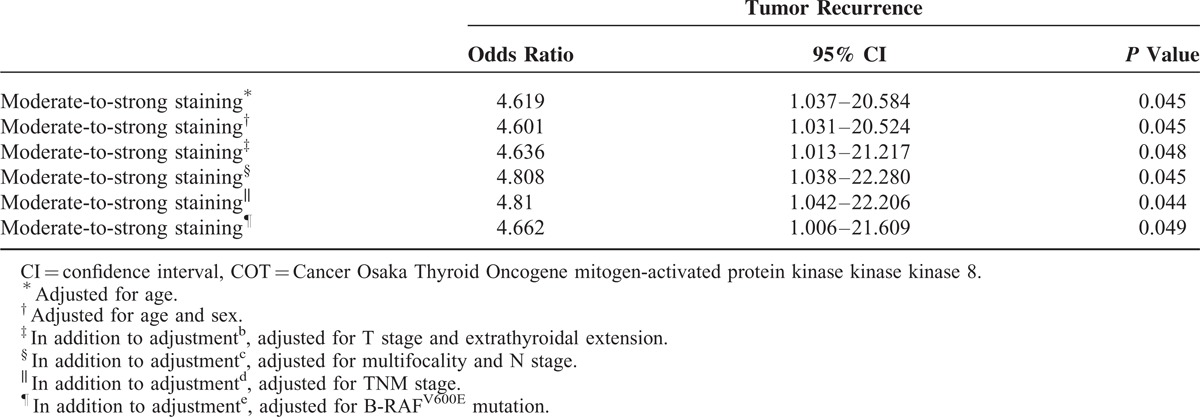
Multivariate Analysis of the Association of Tumor Recurrence With COT Expression Levels

### COT-correlated Genes Indicated by GSEA

**FIGURE 5:**
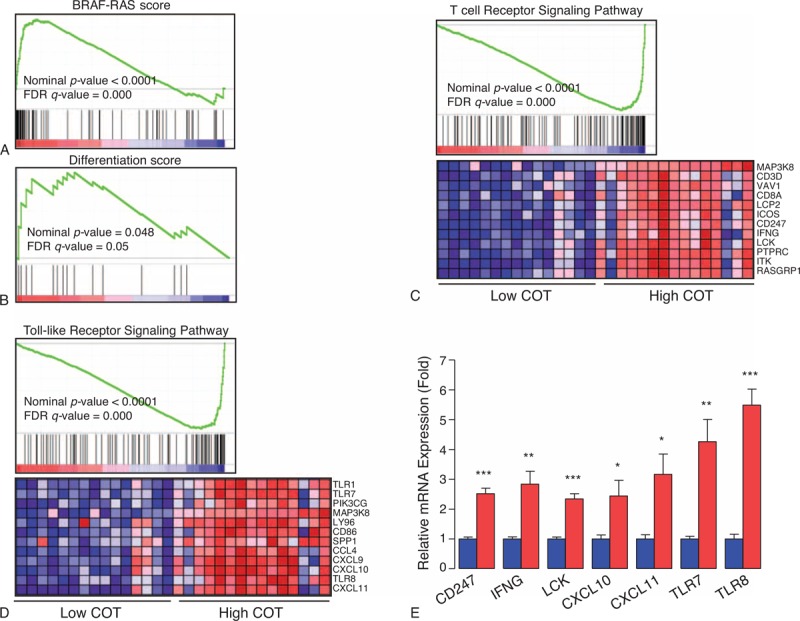
Relationship between expression levels of COT and gene sets. (A) B-RAF-RAS score, (B) thyroid differentiation score, (C) T-cell receptor signaling pathway, (D) Toll-like receptor signaling pathway. Gene set enrichment analysis using gene-expression profiles selected from NCBI GEO Record GSE33630. See detailed description in Methods and Results sections. (E) Quantitative PCR analysis of representative mRNA expression in highest COT expression PTCs (red box, n = 7) and lowest COT expression PTCs (blue box, n = 7) from our study subjects. Means were compared and analyzed by Mann–Whitney *U* test. All data are means ± standard error mean. ^∗^*P* < 0.05, ^∗∗^*P* < 0.01, ^∗∗∗^*P* < 0.001. All *P* values are 2-sided. All experiments were repeated 3 times, and each experiment was performed in triplicate. COT = Cancer Osaka Thyroid Oncogene mitogen-activated protein kinase kinase kinase 8, IHC = immunohistochemical staining, PTC = papillary thyroid cancer.

To get further insight into the molecular biological effects of COT expression, we decided to perform GSEA using data from a public repository, NCBI GEO (GSE33630, total 49 PTC samples). Recently, integrated genomic characterization of PTC suggested that B-RAF-RAS score (BRS) and differentiation score (TDS) can be useful to classify PTC into molecular subtypes.^32^ Using this scoring system, BRAF^V600E^-positive PTC indicated low BRS and TDS. In line with these findings, when we performed GSEA on the PTC samples with the lowest COT expression (n = 15) and those with the highest COT expression (n = 15), we observed the gene sets related to BRS and TDS were coordinately enriched in the lowest COT expression PTCs (Figure 5A and B). In fact, COT has also an important role to activate IKαB kinases, producing nuclear factor-κB.^33^ Furthermore, COT promotes the tumor necrosis factor (TNF)α and interleukin (IL)-2 production for T-lymphocyte activation.^34,35^ Supporting to this previous reports, in our GSEA (Table 3), the top 20 *KEGG* gene sets enriched in the highest COT expression PTCs included T-cell receptor signaling pathway (*P* < 0.0001, false discovery rate [FDR] *q* value = 0.000, Figure 5C) and Toll-like receptor signaling pathway (*P* < 0.0001, FDR *q* value = 0.000, Figure 5D) verified by qPCR using cDNA from our study subjects (Figure 5E). Taken together, our GSEA data suggested that COT might have multifaceted functions in cell proliferation and inflammatory events of thyroid carcinogenesis.

**TABLE 3:**
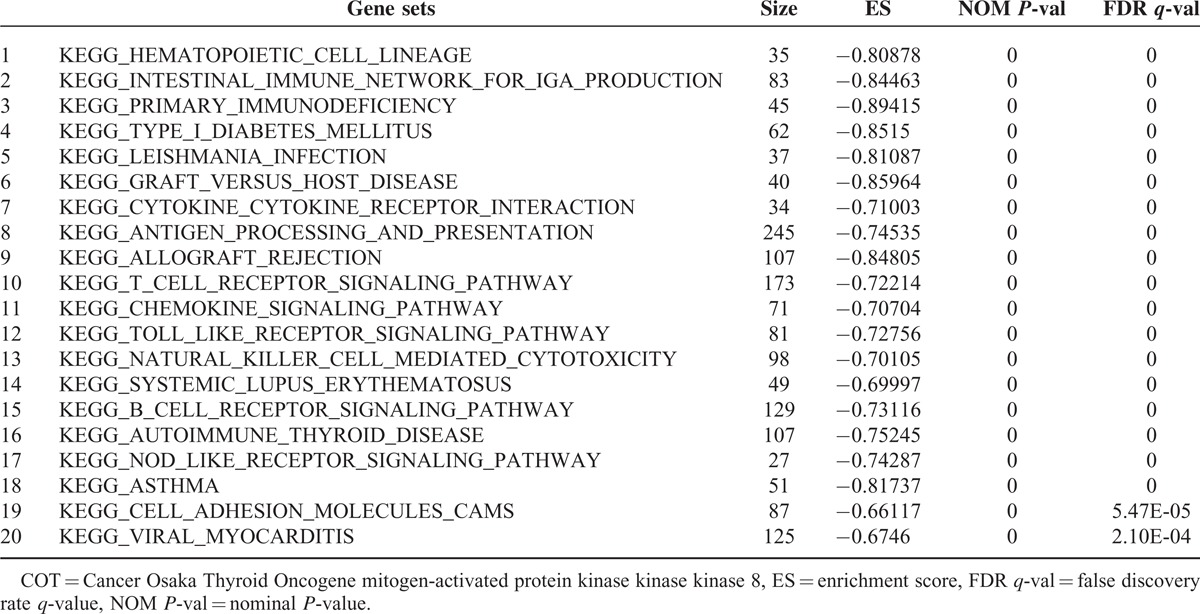
Top 20 KEGG Gene Sets Enriched in Highest COT Expression Group From GSE336300

## DISCUSSION

Following the discovery of B-RAF^V600E^ mutation as an oncogenic kinase in various cancers including melanoma, thyroid, lung, and cholangiocarcinoma, targeted agents against B-RAF^V600E^ kinase have taken a central role in cancer therapy. In this regard, sorafenib has activity against B-RAF^V600E^ and is licensed to treat RAI-refractory PTC.^27^ In addition, other RAF inhibitors such as SB590885, encorafenib, dabrafenib, and vemurafenib are potential therapeutic anticancer agents with clinical utility.^36–39^

In contrast to the high response rate of metastatic melanomas to B-RAF inhibitors, RAF or MEK inhibitors show limited efficacy in RAI-refractory thyroid cancer and thyroid cancer cell lines harboring B-RAF^V600E^.^28,29,40^ One of the possible explanations for the poor response to B-RAF inhibitors in thyroid cancer is related to feedback-induced ligand-dependent activation of HER2/HER3 signaling.^29^ In fact, recent biological and clinical studies have revealed multiple mechanisms of drug resistance: elevated expression of C-RAF, COT1, or mutant BRAF kinases; activating mutations in *N-RAS*, *MEK1*, or *AKT1*; aberrant splicing of BRAF (p61BRAF); activation of phosphatidylinositol-3-OH kinase (PI3K) by phosphatase and tensin homolog (PTEN) loss; and activation of receptor tyrosine kinases, including platelet-derived growth factor receptor, beta polypeptide, insulin-like growth factor 1 receptor, and epidermal growth factor receptor. Interactions between tumors and their microenvironment are also related to innate drug resistance to B-RAF inhibitors.^29^

In this study, qPCR was performed to estimate the expression of A-, B-, C-RAF, and COT mRNAs. Interestingly, expression of the 3 RAF paralogues and COT were all increased in PTC. Supporting this observation, our western blot analysis indicated that the protein expressions of A-, B-, C-RAF, and COT were increased in PTC. Unfortunately, IHC-P using commercially available anti-A-RAF and anti-C-RAF antibodies did not generate reliable data in our hands. The IHC-P for A-RAF did not show any staining intensity and the IHC-P for C-RAF presented nuclear staining, which implicated nonspecific staining because CRAF is a kind of cytosolic proteins. However, our IHC data clearly demonstrated overexpression or aberrant expression of BRAF and/or COT in PTC compared with normal thyroid tissues. In line with our data, inspection of transcriptome profiles indicates that COT expression is increased in certain malignancies compared with normal tissues (http://www.oncomine.org).^35^ Furthermore, the expression of COT showed a strong positive correlation with RAF paralogues, suggesting that this de novo drug resistance mechanism is coordinately regulated in PTC. Taken together, we postulated that de novo drug resistance mechanisms to RAF inhibitors might be active in a significant proportion of PTC with expression of COT.

In IHC-P using anti-BRAF antibody, we could observe focal or moderate staining intensities in normal thyroid tissues suggesting that B-RAF might be required in normal follicular proliferation. In the case of COT, we could observe moderate staining intensity in 2 cases of normal thyroid tissue derived from patients showing high titer of anti-TPO antibody. In these 2 cases, we could also observe lymphocytic infiltration around thyroid follicles. Taken together, we postulated that COT expression might also play a role in inflammatory process such as autoimmune thyroid disease.^41,42^

The other interesting finding is that the B-RAF^V600E^ mutation is related to higher B-RAF or COT expression in PTC. This observation is clinically important because metastatic PTC harboring B-RAF^V600E^ presents RAI nonavidity and suppression of B-RAF/MEK/MAPK pathway is able to restore thyroid specific gene expression for effective RAI therapy.^43,44^ However, in line with our finding, there is a possibility that RAI-refractory PTC harboring B-RAF^V600E^ mutation with increased BRAF and COT expression might present primary drug failure using Sorafenib, which means de novo drug resistance.

Finally, we investigated potential correlations of B-RAF and COT expression with clinicopathological parameters. In the analysis of B-RAF, we could not find any clinicopathological significance. However, because all PTCs showed moderate-to-strong staining intensities in our experiments, we concluded that such kind of analysis is not suitable in this situation. Interestingly, aberrant COT expression has significant impacts on clinicopathological parameters. Higher age at initial diagnosis was correlated with higher COT expression, and importantly higher COT expression was also related to recurrence of PTC. This indicates that COT is related not only to de novo drug resistance, but also to tumor aggressiveness. In fact, we could postulate that COT activates ERK primarily through MEK-dependent mechanisms, resulting in increased ERK-dependent transcriptional output without RAF signaling.^11,45–47^ However, COT has an important function in the signal activator of pro-inflammatory pathways in promoting cancer-associated inflammation of tumor environment.^48,49^ For example, COT has a pivotal role in TNF, IL-1, CD40, Toll-like receptor, and G protein-coupled receptor-mediated MAPK signaling, although the full understanding of the biochemical mechanism that lead to the activation of COT still remains to be elucidated.^35,50^ Supporting this finding, our GSEA indicated that high COT expression in PTC was related to immune-related *KEGG* gene sets. In addition, enhanced nuclear expression of Signal transducer and activator of transcription 3 (STAT3) was observed in c-erbB-2 negative breast cancer with COT overexpression.^51^ The other possible mechanism of COT to affect tumor aggressiveness is the active phosphorylation of Pin1 (PeptidylprolylCis/Transisomerase, NIMA-interacting 1) by COT, increasing cyclin D1 abundance.^52^

In this study, we evaluated the relative levels of A-, B-, C-RAF, and COT mRNAs in PTC and matched normal thyroid tissues. Other drug resistance mechanisms, such as the splice variants of B-RAF, have not been investigated extensively in PTC before treatment with novel targeted agents. We have, however, performed a pilot study in 38 cases to determine the presence of mutations in N-RAS (N-RASQ61) and MEK (exons 3 and 6), which are reported to generate drug resistance in PTC. However, no mutations could be detected (data not shown). In agreement with these results, de novo drug resistance mechanisms should be further investigated in future studies so that unnecessary treatment can be avoided. Furthermore, the role of COT in tumor biology should be focused on improving our understanding of the mechanisms responsible for RAI-refractoriness.

In conclusion, RAF paralogues and COT expression levels are higher in PTCs than in normal thyroid tissues. Aberrant expression of COT correlates with both the BRAF^V600E^ mutation and tumor recurrence. Our data suggest that COT will be an important molecular target for the treatment of RAI-refractory B-RAF^V600E^-positive PTC and for the prediction of tumor recurrence at initial diagnosis.
